# Frequency, Clinical Characteristics, and Management of Snakebite Patients Admitted at the Envenomation Treatment Center of the Applied Biology Research Institute of Guinea

**DOI:** 10.3390/tropicalmed9100238

**Published:** 2024-10-11

**Authors:** Mohamed Ciré Diallo, Karifa Kourouma, Saidou Boumbaly, Armand Saloun Kamano, Abdoulaye Sow, Fassou Mathias Grovogui, Sahar Traore, Alexandre Delamou

**Affiliations:** 1 Institut de Recherche en Biologie Appliquée de Guinée (IRBAG), Kindia 00224, Guinea; diallomto@gmail.com (M.C.D.); boumbalys@gmail.com (S.B.); sahartra1900@gmail.com (S.T.); 2 Centre National de Formation et de Recherche en Santé Rurale de Maferinyah (CNFRSR), Maferinyah, Forécariah B.P. 2649, Guinea; askamano@maferinyah.org (A.S.K.); mgrovogui@maferinyah.org (F.M.G.); adelamou@cea-pcmt.org (A.D.); 3 Public Health Department, Gamal Abdel Nasser University of Conakry, Conakry B.P. 1147, Guinea; drsowab@msn.com; 4 African Center of Excellence for the Prevention and Control of Communicable Diseases (CEA-PCMT), Gamal Abdel Nasser University of Conakry, Conakry B.P. 1017, Guinea

**Keywords:** snakebite, management, Guinea, neglected tropical diseases

## Abstract

The aim of this study was to describe the frequency, clinical signs, management, and outcomes of snakebite patients admitted to the envenomation treatment center of the Institut de Recherche en Biologie Appliquée de Guinée (IRBAG). This was a retrospective review combining aggregated annual statistics (2011–2015) and routine data (from January to October 2021) from the IRBAG treatment center. There were 1345 (57.2%) snakebite victims out of a total of 2352 consultations at the center during the study period. Males (67.7%), persons aged ≥45 years (29%) and ≤14 years (27.7%), farmers/housewives (44.5%), workers (23.9%), and those residing in the Kindia Prefecture (53.5%) were the most commonly affected. The majority of victims (84.5%) were admitted three hours after snakebite, with bites mainly occurring in rural areas (86.5%) and during the rainy season (83.2%). Pain (100%), edema (76.8%), and bleeding (65.2%) were the most common clinical presentations. Almost all victims received antivenom serum (98%), antibiotics (87.7%), and analgesics or anti-inflammatory drugs (88.4%). Six out of the one hundred and fifty-five patients died. Snakebites are a frequent public health problem in rural Guinea. The majority of victims seek medical attention too late. There is an urgent need to include snakebite in the country’s list of priority NTDs in order to promote access to antivenom serum.

## 1. Introduction

Snakebite is one of the neglected tropical diseases (NTDs) that constitute a major public health problem, particularly in resource-limited countries [1]. The World Health Organization (WHO) estimates that around 5.4 million people worldwide suffer from snakebites every year and these result in 400,000 disabilities and more than 125,000 deaths [2]. Sub-Saharan Africa is said to bear the burden of snakebites, with mortality ranging from 3000 to 32,000 deaths per year, mainly affecting people living in rural and poor areas [3]. This high mortality rate is associated with factors such as late access to healthcare services and a lack of antivenom at the health facilities [3]. In response to this state of affairs, the 6th International Conference on Envenomation, held in Africa in 2015, recommended improving the epidemiological information (through routine data), better envenomation case management, and universal access to antivenom [4].

Since then, studies in Africa have reported on the epidemiology and factors associated with snakebites [5]. For example, in Nigeria, a review of data from people with snakebites from a teaching hospital reported that 82% of young adults aged 15–40 years were the most commonly affected, with half showing signs of envenomation [6]. Ochola and colleagues in Kenya also reported a high incidence of snakebites, especially during the dry season [7].

In Guinea, there is a limited amount of data available on snakebites, and it is concerning that the National Program for the Control of Neglected Tropical Diseases (NPNTD) does not currently prioritize snakebite as a neglected tropical disease [8]. The only recent research related to snakebites in Guinea focused on the evaluation of the efficacy and tolerance of antivenoms such as “Antivipmyn Africa” [9], “Pan-African Inoserp” [10], or the use of ketamine to alleviate pain in victims [11]. However, to date and to our knowledge, no antivenoms are available at primary public healthcare facilities (hospitals and health centers) to support the management of snakebite patients. Furthermore, the only available data on the clinical characteristics of snakebite patients in Guinea comes from a single case study involving a double snakebite [12]. Additionally, there have been no studies conducted on medically significant snake species within the country to guide the administration of antivenoms where available. Therefore, in order to advocate for the inclusion of snakebites in the priority list of neglected tropical diseases and to provide guidance for future interventions, it is necessary to generate epidemiological data that demonstrate the extent of snakebites in Guinea. Consequently, this study aimed to provide a comprehensive analysis of the frequency, clinical characteristics, management, and outcomes of snakebite patients admitted to the envenomation treatment center at the Applied Biology Research Institute of Guinea (IRBAG). Additionally, this study was conducted within the framework of the initial implementation of a structured operational research training initiative, primarily aimed at generating data that could inform interventions by the Guinean national program for neglected tropical diseases due to the scarcity of existing data on snakebites in the country.

## 2. Materials and Methods

### 2.1. Study Design

This was a retrospective review combining aggregated annual statistics (2011–2015) and routine data (from January to October 2021, 155 snakebites victims) from the IRBAG. Moreover, the choice of the present study design and period was based on the fact that no detailed records (individual patients’ data) were kept for the period 2011–2015.

### 2.2. Study Setting

#### 2.2.1. General Setting

Guinea is a West African country with an estimated population of over 13 million in 2022, the majority (70%) of whom live in rural areas [13]. The national public health system comprises three levels of care: primary (407 health centers and 1640 health posts), secondary (9 communal medical centers, 7 regional hospitals, and 26 prefectural hospitals), and tertiary (3 national hospitals).

#### 2.2.2. Specific Setting

The Kindia Prefecture, located 135 km from the capital Conakry, had a population of 554,224 in 2022. The majority live in rural areas, with agriculture as their main source of income [13]. The district comprises 11 sub-prefectures plus the urban center (Kindia city). The prefecture’s local health system includes a regional hospital, five health centers, and seven health posts. Geographically, Kindia district is a transition zone linking the capital, Conakry, to the hinterland, with a geographical mix of plains and high plateaus [14].

The IRBAG envenomation treatment center, located in Kindia district, specializes in the treatment of snakebites in the Lower Guinea region. Created and launched in 1922, the center comprises five departments, Venerology, Virology, Zoology-Medical, Bacteriology, and Clinical Epidemiology) and receives over 500 consultations per year. The Center is staffed by two doctors and three nurses. Additionally, it also possesses a production capacity of snakebite antivenom primarily for research purposes.

### 2.3. Study Population and Period

All snakebite patients admitted at the envenomation center at the IRBAG during the study period constituted our study population.

### 2.4. Data Sources and Collection

Data were collected from patient files and consultation registers using a pre-designed Excel questionnaire (version 2019) as well as the IRBAG’s aggregated annual statistics. A team of three research assistants helped to collect the data under the supervision of the principal investigator.

### 2.5. Data Management and Analysis

Data were processed in Excel and then exported to Stata version 17 (Stata Corporation, Collège Station, TX, USA) for analysis. Results were presented in descriptive form using frequencies, proportions, medians, and interquartile ranges.

### 2.6. Ethical Considerations

The protocol for this study was approved by the National Ethics Committee for Research in Health of Guinea (No. 139/CNERS/22). In the field, authorization from the IRBAG authorities was obtained before accessing the data. As this was a retrospective review of routine data, a waiver for obtaining informed consent from snakebite patients was obtained.

## 3. Results

**Figure 1:**
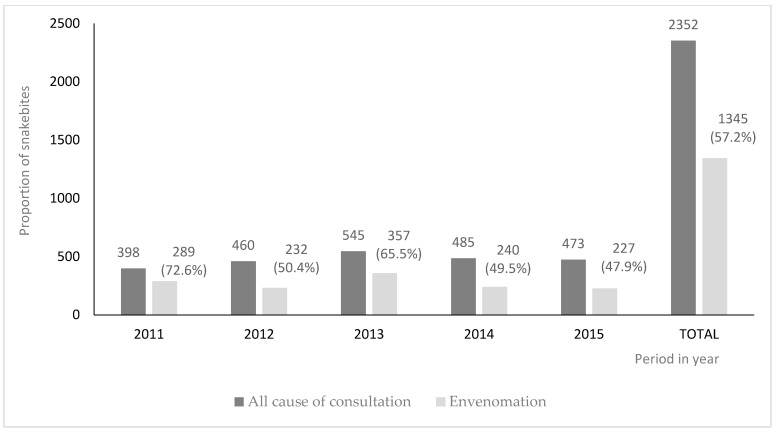
Frequency of annual consultations (all causes combined) and those due to snakebites at the IRBAG envenomation treatment center in Kindia, Guinea, 2011–2015.

Based on the aggregated annual statistics from the IRBAG, snakebite accounted for more than half (57.2%; 1345/2352) of all consultations (Figure 1). Table 1 presents the socio-demographic characteristics of snakebite victims based on individual routine data from 2011 to 2015. The most commonly affected groups were males (67.7%), individuals aged ≥45 years (29%) and ≤14 years (27.7%), farmers/housewives (44.5%), workers (23.9%), and those residing in Kindia district (53.5%).

Table 2 presents the clinical characteristics and outcomes of snakebite victims admitted to the IRBAG envenomation treatment center. A majority of the victims (84.5%) were admitted to the treatment center more than three hours after the snake bite occurred. The bites mostly occurred in rural areas (86.5%) and during the rainy season (83.2%). Pain (100%), edema (76.8%), and bleeding (65.2%) were the most common presenting clinical signs. Almost all victims received antivenom (98.1%), antibiotics (87.1%), and painkillers (88.4%). Out of the one hundred and fifty-five patients, six died.

## 4. Discussion

Our study conducted a thorough investigation into the frequency, clinical characteristics, treatment, and outcomes of snakebite cases in Guinea. It is worth noting that our study belongs to a limited number of studies that have explored this topic in depth. The results of our study highlighted a higher frequency of snakebites among children under the age of 14 and individuals aged 45 and above. Furthermore, we found that males, those residing in rural areas, farmers, housewives, and workers in the Kindia Prefecture were more vulnerable to snakebites. Moreover, our data revealed that a significant number of snakebite victims sought medical care at a late stage and displayed symptoms such as pain, swelling, and bleeding.

However, it is important to acknowledge the limitations of our study. The retrospective design and small sample size hampered our ability to thoroughly investigate factors associated with mortality in snakebite victims. Additionally, due to the study design, we were unable to assess long-term complications resulting from snakebite envenoming, the treatment-seeking behavior of patients (e.g., whether they sought care from pharmacies, clinics, traditional healers, etc.), and the length of hospital stay. Moreover, the ten month duration covered by the routine data utilized in our study may not provide a comprehensive understanding of the true burden of snakebites in Guinea. Therefore, future research should address these gaps to comprehensively assess the impact of snakebites in the country. Despite these limitations, our study holds implications that can be inferred.

Firstly, the frequency of snakebites among children under the age of 14 and adults aged 45 and above living in rural areas, particularly among farmers, aligns with findings from other studies conducted in Rwanda, Cameroon, Congo, Togo, and the Central African Republic [15,16,17,18,19]. The higher frequency of snakebites among adult farmers can be attributed to their daily activities involving vegetation clearance and the handling of seed products, which increases their direct exposure to snakes. The increased occurrence of snakebites among children can be attributed to their involvement in assisting their parents in field-related tasks and their immature and careless behavior while playing in the fields. It is crucial to raise awareness among rural populations, especially farmers, regarding the importance of using appropriate protective equipment such as boots and durable plastic gloves, and the need to supervise children to minimize the risk of snakebites.

Secondly, the majority of snakebite victims in our study presented late in seeking treatment at the IRBAG envenomation treatment center, displaying pain, swelling, and bleeding as the primary clinical indications. Consistent with previous research, our findings demonstrate that edema and bleeding are the predominant symptoms [15,17,20]. However, the timeframe between the occurrence of the snakebite and medical attention in our study was longer compared to a study conducted in Cameroon (ranging from 45 min to 2 h) [16], but notably shorter than that reported in Togo (≥24 h) [19]. In our particular context, various factors likely contributed to the delayed access to healthcare services. These factors may include the population’s heavy reliance on traditional medicine (such as the application of black stone, tourniquet, or incision at the bite site), consequently leading to a delay in seeking proper medical care and an increased vulnerability to complications and mortality for the victims [16]. Compounding this issue are hurdles in reaching healthcare services due to geographical distance, the prevailing poverty among rural communities (living below the national poverty line of USD 2 per day), and the unavailability of antivenom serums at primary health facilities (e.g., health posts and centers). By improving the availability of antivenoms in primary healthcare facilities and simultaneously enhancing awareness among the community and traditional healers regarding the importance of promptly referring snakebite victims to health facilities, we can augment prognosis and prevent long-term disabilities among snakebite victims.

Thirdly, nearly all snakebite victims in our study received antivenom serum, a contrast to findings from studies conducted in Rwanda (8%) and Cameroon (2%) [16,17]. The greater proportion of snakebite victims receiving antivenom in our study can be attributed to the fact that all of these patients were admitted to the specialized IRBAG envenomation treatment center, which possesses the production capacity of snakebite antivenom, testing, and management of snakebite patients in the country.

Fourthly, our data unveiled a low mortality rate among snakebite victims, comparable to the results from studies carried out in the Central African Republic [15] and Togo [19]. However, caution should be exercised when interpreting this low mortality rate, as accurate individual data were only obtained for a total of 155 snakebite patients during the study period, possibly resulting in an underestimation of the true mortality rate. Finally, this study illustrates the significance of snakebites and the imperative to integrate this overlooked tropical disease into Guinea’s national strategic plan for addressing such illnesses. Moreover, the data draw attention to the obstacles associated with the availability and accessibility of antivenom at primary healthcare facilities, specifically in rural regions. A comprehensive approach is essential, encompassing the establishment of standardized protocols for overseeing snakebite patients and the enhanced engagement of local communities at every level.

## 5. Conclusions

Snakebites pose a significant public health challenge in rural Guinea, occurring with high frequency. Unfortunately, the majority of individuals affected by snakebites delay seeking medical treatment. It is therefore crucial to prioritize snakebite management as a part of the country’s national agenda for neglected tropical diseases, thereby improving the access to antivenom serum, especially at primary health facilities.

## Figures and Tables

**Table 1:** Socio-demographic characteristics of snakebite patients admitted to the IRBAG envenomation treatment center in Kindia, Guinea, 2011–2015 (N = 155).

Characteristics	N	Percentage
Age (in years)		
*≤14*	43	27.7
*15–29*	35	22.6
*30–44*	32	20.6
*≥45*	45	29.0
Median age (IQR)	28 (14–45)	
Gender		
*Male*	105	67.7
*Female*	50	32.3
Occupation		
*Farmer/housewife*	69	44.5
*Pupils/students*	37	23.9
*Manual workers*	37	23.9
*Others* ^a^	12	10.3
Residence		
*Kindia*	83	53.5
*Mamou*	13	8.4
*Dubreka*	13	8.4
*Others* ^b^	46	29.7

**IRBAG:** Guinean Research Institute of Applied Biology. **IQR**: Interquartile range. ^a^ **Others**: Children < 5 years (10), tradesmen (2). ^b^ **Others:** Télimélé (8), Gaoual (6), Lélouma (6), Pita (5), Dalaba (4), Dinguiraye (3), Forécariah (3), Boffa (2), Kouroussa (2), Labe (2), Boke (1), Conakry (1), Siguiri (1), Coyah (1), and Fria (1).

**Table 2:** Clinical characteristics and outcomes of snakebite victims admitted to the IRBAG envenomation treatment center in Kindia, Guinea, 2011–2015 (N = 155).

Variables	N	Percentage
Time between snakebite and admission (in hours)		
1–3	24	15.5
*>3*	131	84.5
*Median time between snakebite and admission (IQR)*	4.0 (1.9–6.1)	
Place of bite		
*Rural*	134	86.5
*Urban*	21	13.5
Season when snakebite took place		
*Dry*	26	16.8
*Rainy*	129	83.2
**Symptoms**		
*Pain*	155	100.0
*Vomiting*	29	18.7
*Sialorrhea*	20	12.9
*Dyspnea*	16	10.3
*Dysphagia*	25	16.1
*Palpebral ptosis*	24	15.5
*Mouth paralysis*	18	11.6
*Edema*	119	76.8
*Bleeding*	101	65.2
*Blisters*	17	10.9
*Others* *	16	0.1
Bite sites		
*Hands*	22	14.2
*Feet*	129	83.2
*Others ^λ^*	4	2.6
Administered treatments		
*Intravenous solutions*	23	14.8
*Anti-inflammatory drugs/analgesics*	137	88.4
*Antibiotics*	135	87.1
*Antivenom Serum*	152	98.1
Outcomes		
*Discharge*	147	94.8
*Died*	6	3.9
*Referral to another center*	2	1.3

* **Others:** Myosis (1), dysgeusia (1), diarrhea (2), epigastric pain (3), headache (2), tingling (7). ***^λ^* Others:** Head (2), sex (1), eye (1).

## Data Availability

The database used in this study is available on request from the corresponding author.
